# Faithful Experimental Models of Human *Mycobacterium Tuberculosis* Infection

**DOI:** 10.4172/2161-1068.1000e108

**Published:** 2012-02-20

**Authors:** Deepak Kaushal, Smriti Mehra

**Affiliations:** Tuberculosis Research Program, Division of Bacteriology & Parasitology, Tulane National Primate Research Center, Covington, LA, 70433, USA

In spite of the remarkable progress made by the medical science in the last century, the effectiveness of tuberculosis (TB) treatment and vaccination has remained less than satisfactory. This has placed a very high burden on the public health systems of the third world. Annually, about 1.5 million deaths are attributed to TB [1]. The number of new cases seems to be on the rise, topping 9 million in the recent years [1]. Co-infection with HIV-AIDS has further worsened the global TB situation [2]. A further concern is the advent of drug resistant (including multi- and extensively-drug resistant) strains of *Mycobacterium tuberculosis* (*Mtb*) [3].

As a result, TB remains one of the top three infectious disease killers of mankind. Our inability to control TB stems from the relative lack of new and effective anti-TB drugs and vaccines.

This in turn, is a testament to the highly successful and specialized pathogenic nature of *Mtb*. While important strides have been made in understanding the basis of virulence and pathogenesis of TB, clearly, more needs to be done.

One of the most interesting characteristics of *Mtb* infections is the ability of the pathogen to reside in a latent (or dormant) state for months, years and perhaps decades (latency) [4]. *Mtb* infections induce the formation of granulomatous lesions in the lung. The granuloma is a hallmark of an *Mtb* infection, although some other infections also induce its formation [4]. The granuloma consists of both innate and adaptive immune cells, and represents an attempt by the host to wall off the initial foci of infection. However, it is now believed that the tubercle bacilli actively promote granulomatous pathology, since it has the specialized machinery to adapt to survive in an intra-granulomatous environment in a latent state. These dormant bacilli retain the ability to recrudesce at a later time when the immune system is compromised, e.g. by HIV co-infection or old age (reactivation) [4]. Moreover, prior *Mtb* infection does not lead to sterilizing protection and latently infected individuals remain susceptible to reinfection [5].

While *in-vitro* studies have extended our knowledge of the pathogenesis of TB, we still don’t conclusively understand the molecular and immunological basis of latency and reactivation. Furthermore, the complete identity of correlates of protection, as against correlates of disease progression, is not known. This is compounded by the fact that the human TB syndrome consists of a spectrum of different conditions, governed by host immunity, pathogen virulence and a number of environmental variables [6].

Since human beings are the only known natural host of *Mtb*, mechanistic insights into the process of *Mtb* infection and pathogenesis require the use of robust experimental animal models that faithfully represent the various aspects of human TB. Since the time of Koch [7], animal models have been employed with a great deal of success to study *Mtb* infection. Mice, guinea pigs, rabbits, fish, cattle and nonhuman primates have all been used to model TB [8].

The mouse is the most extensively used model of TB, due to its ease of use and inexpensive nature. Moreover, the availability of genetically pure transgenic or knockout strains of mice allow defined mechanistic studies into the role of key immune molecules e.g. TNFα and IFNγ. Molecular, cellular and immunological reagents for a wide-variety of assays are easily available since mice are the choice lab animal models. However, several important factors detract from the usefulness of the mouse model: i) Mice are unable to truly model latency from the standpoint of either the pathogen or the host; ii) Mice do not display variable progression following experimental infection, characterized by a spectrum of experimental conditions, ranging from asymptomatic infection to fulminant, acute disease; iii) the entire range of pathological lesions found in the lung tissues of infected humans is not reproduced in the mouse model [8]. It is believed that the human pulmonary granulomatous lesions subject *Mtb* to a wide variety of stress, including hypoxia, lack of essential nutrients etc. This likely has a fundamental impact on the physiology as well as metabolism of *Mtb*. In fact, it has been suggested that studies the absence of these stress conditions do not subject may yield flawed results [9]. Guinea pigs suffer from extreme susceptibility to *Mtb* infection and are thus unable to replicate latency. The rabbit model lacks defined molecular and immunological reagents.

In the past decade, nonhuman primates, in particular, rhesus (*Macaca mulatta*) and cynomolgus (*Macaca fascicularis*) macaques have garnered interest due to their abilities to better model different aspects of human TB [10]. Based on the dose presented as well as the virulence of the strain, these animals generate either latent [11,12] or acute TB [12–14]. Moreover, latent TB in these animals can be reactivated significantly by either co-infection with Simian Immunodeficiency Virus (SIV), which is closely related to HIV, or via TNFα neutralizing therapy [11,15,16]. BCG-vaccination prior to *Mtb* exposure induces partial protection, again mirroring the human infection [17].

Another advantage of the NHP model is that clinical correlates of the infection, including thoracic X-rays, tuberculin skin test, Blood CBC and chemistries etc. are easily performed. Further, the NHP model allows the sampling of the same animal repeatedly, including prior to infections. Therefore, it is possible to sample lung biopsies or bronchoalveolar lavage at different time-points post-infection for techniques like RT-PCR, microarrays, RNAseq, confocal multilabel microscopy and flow-cytometry, and control these experiments to the pre-infection sample from the same animal. At the same time, readouts of *Mtb* colony forming unit load, histopathology of granulomatous lesions and clinical measures of disease progression can also be obtained, allowing a multi-parametric analysis of host-pathogen interactions.

Other advantages of the NHP model include the wide availability of molecular and immunological reagents. Many human antibodies and reagents react to monkey epitopes. Moreover, antibodies and inhibitors against key immunological markers of Th1, Th2, iTreg and Th17 signaling pathways are available for NHPs due to their long history of use in AIDS research. In the last decade, the entire genome sequence for *M. mulatta* and several other NHPs has become available. It has been possible to employ state of the art methods such as transcriptomics [18], computed tomography scanning [19] and positron emission tomography [20], to this model.

Clearly, infections in NHPs closely model human TB. In spite of its expense and the requirement of specialized laboratory and housing space, this model is critical for both, a better understanding of *Mtb* pathogenesis as well as for a true evaluation of anti-tubercular drugs and vaccines.

As discussed earlier, the oft-used conventional mouse model does not represent the true granulomatous pathology associated with human disease. However, a mouse model has been recently introduced where necrotic, caseous lung lesions are generated [21]. These caseous lesions have recently been shown to be hypoxic in nature. Popularly known as the “Kramnik mice”, these animals harbor the C3HeB/FeJ genotype. In addition to the lesions being pathologically caseous, the *Mtb* contained in them also express the members of the hypoxia/microaerophilia-induced DosR regulon. Further, treatment with drugs thought to be effective under hypoxic conditions rapidly cleared *Mtb* infection in this model [21]. These results suggest the potential of genetics in evolving the current mouse model of TB to better represent aspects of the human TB syndrome.

The nonhuman primate and the “Kramnik mouse” models of TB represent two experimental systems that faithfully represent key aspects of human TB. These models have the potential to allow us to validate novel candidate drugs and vaccines. Further, we expect elusive mechanisms of pathogenesis of *Mtb* may be revealed through the use of these model systems.
